# Co-producing Randomized Controlled Trials: How Do We Work Together?

**DOI:** 10.3389/fsoc.2019.00021

**Published:** 2019-03-29

**Authors:** Lucy Pollyanna Goldsmith, Rosaleen Morshead, Charlotte McWilliam, Gordon Forbes, Michael Ussher, Alan Simpson, Mike Lucock, Steve Gillard

**Affiliations:** ^1^Population Health Research Institute, St George's, University of London, London, United Kingdom; ^2^Pragmatic Clinical Trials Unit, Queen Mary University of London, London, United Kingdom; ^3^Institute of Psychiatry, Psychology & Neuroscience, and Florence Nightingale Faculty of Nursing, Midwifery & Palliative Care, King's College London, London, United Kingdom; ^4^School of Human and Health Sciences, University of Huddersfield, Huddersfield, United Kingdom

**Keywords:** coproduction, randomized controlled trial (RCT), quantitative research approaches, reflective practice, methodology and methods of sociological research, peer support (PS)

## Abstract

In the light of the declaration “Nothing about us without us” (Charlton, 2000), interest in co-production, and coproduced research is expanding. Good work has been done establishing principles for co-production (Hickey et al., 2018) and for good quality involvement (Involve, 2013; 4Pi, 2015) and describing how this works in practice in mental health research (Gillard et al., 2012a,b, 2013). In the published literature, co-production has worked well in qualitative research projects in which there is often methodological flexibility. However, to change treatment guidelines in the UK, e.g., the National Institute for Health and Care Excellence guidelines, and influence service commissioning, high quality quantitative research is also needed. This type of research is characterized by formal methodological rules, which pose challenges for the scope of co-production. In this paper we describe the significant challenges and solutions we adopted to design and deliver a coproduced randomized controlled trial of mental health peer support. Given the methodological rigidity of a randomized controlled trial, establishing clearly which methodological and practical decisions and processes can be coproduced, by whom, and how, has been vital to our ongoing co-production as the project has progressed and the team has expanded. Creating and maintaining space for the supported dialogue, reflection, and culture that co-production requires has been vital. This paper aims to make our learning accessible to a wide audience of people developing co-production of knowledge in this field.

## Introduction

We discuss co-production in quantitative research (with a specific focus on randomized controlled trials), how it can work in practice, and the barriers and enablers of co-production. We understand co-production in research in terms of a knowledge framework. Using standpoint epistemologies familiar to feminism (Harding, 1991) and other critical disciplines, the active involvement of people who have made use of healthcare services as researchers, brings a service user knowledge (Beresford, 2013), or experiential knowledge (Rose, 2017), critiquing and challenging dominant and universalizing ways of knowing about health, with a primary objective of democratizing research (Pinfold et al., 2015). This experientially grounded, or tacit knowledge, acquired through private and personal ways of knowing the mind and body, differs from a more codified or theoretical knowledge acquired through study and training (Mol and Law, 2004), and offers a competing discourse in a healthcare context (Nowotny et al., 2001; Mockford et al., 2012). A definition of co-production in the context of multidisciplinary mental health research involving people who have used mental health services as members of the research team is given in Box 1 below.

Box 1 **Co-production (Gillard et al., 2012a).**

1. High-value research decision-making roles distributed across the research team.
2. Different interpretations of data within the research team owned and understood in terms of how who we are has shaped the knowledge we have produced.
3. Consideration given to whether all members of the team were involved in the production of knowledge throughout the research project and the impact of this considered.
4. Methodological flexibility allowed in the research process where scientific conventions constrains the input of particular team members.
5. Rigorous and critical reflection on why the research was done in the way it was as integral to the conduct of the research.
6. Research outputs that report critically on how knowledge was produced.

Roper et al. (2018) outline three core principles of co-production in the context of mental health research. First “consumers being partners from the outset” means service users should be involved in all aspects of the research process from the beginning and be privy to all information. Professionals implicitly or explicitly valuing the knowledge of professionals over lived experience (Scholz et al., 2017), or equating people who use mental health services with their diagnoses or symptoms, may hamper their ability to perceive the value in what service users say (Happell et al., 2015; Kopera et al., 2014). The second principle states power differentials and tokenism must be “acknowledged, explored and addressed.” Power differentials exist within the relationship between professionals and service users and present a challenge to genuine co-production (McDaid, 2009; Rose et al., 2010). Whilst is it suggested that co-production helps transform relationships, co-production can create power sharing risks that will lead to both parties (professionals and people who use services) feeling uncomfortable at times. For those with lived experience, the power differentials can echo disempowering experiences of using services, resulting in a reluctance to coproduce (Lwembe et al., 2016). Reluctance to coproduce can also be experienced by professional researchers. One strong description of the demands placed on researchers by co-production is that they are required to renounce their privileged position as sovereign experts and their monopoly on scientific knowledge and step down from the proverbial ivory tower of the academy to enter into dialogue with the world around them (Phillips, 2009). An exploratory study of Recovery Colleges found a willingness on the part of the professionals working in mental health to embrace co-production and relinquish the traditional power differences (Dalgarno and Oates, 2018). Roper et al. (2018) third core principle of co-production is the need to encourage and provide the means for people who use mental health services to take leadership and develop their capacity within services and research. In practice, this is difficult to achieve as there are often insufficient resources to allow full participation, and welfare benefits can be compromised, acting as a barrier to co-production (Lambert and Carr, 2018). Scholz et al. (2017) identifies unclear roles within a structure and a resistance to viewing people who use services as leaders as barriers to leadership.

Co-production of data analysis needs further development as this is currently unusual (e.g., Jennings et al., 2018). There is potential for service user-, clinical-, and academic researchers to coproduce a richer, integrated analytical narrative through challenging the previously taken for granted researcher assumptions and cultural perspectives, producing a more thorough examination of the data (Tuffrey-Wijne and Butler, 2010). In a qualitative study, additional themes were found in the data by service user researchers who coded data in terms of experience and emotions rather than the procedures and processes typically coded by university researchers (Gillard et al., 2012b). Despite the difficulties highlighted, studies cited demonstrate that co-production can work well in qualitative research projects. There is a relative lack of studies reporting attempts to co-produce quantitative research, where there are likely to be additional challenges. We discuss randomized controlled trial methodology and review the critical literature in this area before discussing co-production in the context of randomized controlled trials. Within quantitative research, a randomized controlled trial (RCT) is considered the “gold standard” (Barton, 2000) as randomization reduces the risk of confounding (where the observed effect is due to an inseparable mix of the treatment effect and other effects). Randomized controlled trials generally use blinding of treatment allocation (for researcher) or double blinding (for researcher and participant) to eliminate bias (in which a belief in the therapy can also affect the outcome). Efforts are made to ensure that interactions with the researcher do not have an effect on the outcome, although the effect of the researcher on the research process or participants is not usually measured. Commonly, the researcher attempts to be an impartial observer, and their emotions are not considered relevant to the research [i.e., an “objectivist” approach (Ratner, 2002)]. The main analysis is pre-specified to avoid bias. Research teams can be hierarchical as particular members of the team provide expert authority in aspects of the research (e.g., statistical, clinical, research governance).

Randomized controlled trial methodology has been criticized for producing misleading results (e.g., poor quality trials produce inflated treatment effect estimates; Moher et al., 1998). For trials of complex interventions (in which the treatment contains a mix of interacting elements, e.g., talking therapies, residential interventions, social support), appropriate questions go beyond “does it work” to probe the underlying mechanisms of how, why, for whom and under what conditions interventions work (MRC, 2008; Blackwood et al., 2010). Randomized controlled trials used in health research typically fall into the area of “evidence based medicine,” aiming to mobilizing research to inform healthcare. However, Greenhalgh et al. (2015), for example, argue that evidence based medicine may inadvertently devalue the patient and carer agenda by: (i) limiting patient input into research design; (ii) giving low status to individual experience in the hierarchy of research evidence; (iii) minimizing or ignoring power imbalances that suppress patient voice; (iv) focusing on people who seek and can access care thus ignoring those who do not access services; (v) overlooking the importance of the patient-clinician relationship; (vi) underestimating the value of self-management and lay networks of support.

In summary, co-production can deliver research which incorporates the perspectives of service users and other non-traditional members of the research team; fundamentally changing the knowledge production approach, offering social accountability, and a richer analysis of data, possibly generating conclusions with more relevance to service users. The challenges to working meaningfully in this way are substantial, including tokenism, power differentials, the need for emotional, and practical support for service users. Randomized controlled trials are a powerful tool for finding out “what works” in mental health services, yet the methodology has been criticized in a number of ways, including unrealistic expectations that the research process itself has no effect while suppressing knowledge from lived experience. These criticisms suggest co-production might improve, rather than weaken the randomized controlled trial methodology. For example, some of the challenges in trials (especially of complex interventions with a social component) might be usefully addressed by integrating other types of expertise—including patient and carer insight—into the research process. We identified no prior publications exploring these potentials in a randomized controlled trial. To address this, this paper reports the possibilities and challenges of coproducing a randomized controlled trial.

### Setting

The setting for the research is multisite randomized controlled trial of peer support for discharge from inpatient to community mental health services in the UK (Gillard and Marks, 2016). The trial aimed to recruit 590 participants, randomized 50:50 to peer support and care as usual. The trial hypothesized that participants receiving peer support would be less likely to be readmitted to inpatient psychiatric care in the year post-discharge than participants receiving care as usual. Peer support was provided individually by peer workers—people with previous experiences of using mental health services—selected and trained to provide peer support for the discharge transition and supervised by an experienced peer worker. We note that peer workers did not occupy any other roles within the project (e.g., they were not also researchers).

### Research Team

The research was undertaken by a research team that included a Chief Investigator (a health services researcher), four clinical academics (a psychiatrist, two psychiatric nurses and a clinical psychologist), three statisticians, a health economist, a health psychologist, two managers of peer support services (one working in the NHS and one in the not-for-profit sector), an experienced peer worker (working in the NHS) and two experienced service user researchers. A trial manager who also brought experience of having used mental health services, and a total of nine further service user researchers joined the team to undertake recruitment of participants and data collection. The aim was to coproduce throughout the study, using the framework cited above as a starting point (Gillard et al., 2012a), with all members of the research team involved in the initial conception of the research and the extended team (including trial manager and all service user researchers) involved in decisions made about conducting the trial (i.e., how to put the trial protocol into action) once the research programme was underway.

## Method

Two methods were used to explore co-production in the trial. First, members of the team co-authoring the paper selected examples of decision-making about the design of the trial. We select examples which, to a greater and lesser degree, include a range of members of the research team, and service user researchers working on the trial in the decision-making process (i.e., where there was more or less co-production involved). We illustrate the decision-making process by citing directly from minutes of the different meetings that collectively, manage the trial. These include: the Trial Management Group (TMG—a subgroup of people involved in managing the trial who meet on a monthly basis including Chief Investigator, Senior Trial Statistician, Trial Manager, one of the two experienced service user researchers, Health Economist, the academic psychiatrist plus a data management and a quality assurance advisor); the Lived Experience Advisory Panel (LEAP—a group of people independent from the trial who have personal experience of peer support, using mental health services and working as service user researchers who meet biannually to advise on conduct of the trial from a lived experience perspective); and investigator meetings (large biannual meetings where the whole research team come together). We reviewed those minutes to identify how, when and why the decisions which shaped the scope and focus of the research were reached and the potential impact of those decisions on the research process and outputs (extracts of these are quoted and labeled as “minutes” in the text below).

Second, members of the team who were involved in either making those decisions or implementing them into practice wrote reflections on the decision-making process and consequences (extracts of these are labeled “reflections” where quoted below). Drawing on a sequential method of analysis (Simons et al., 2008), accounts of these meetings and our reflections were iteratively co-edited by the authors as this paper was written. In this sense our shared writing and re-writing was an integral part of our method of enquiry (Richardson, 2000; Simons et al., 2008).

## Findings

We present three key decisions at which co-production was most challenged and/or most productive. We focus on these to illustrate the different perspectives and how these were discussed and resolved. We then reflect on the implications of the decisions made. These decisions relate to: (i) Identification of trial population; (ii) Choice of psychometric measures and outcomes; (iii) Development of the trial statistical analysis plan.

### Trial Population

**Figure 1:**
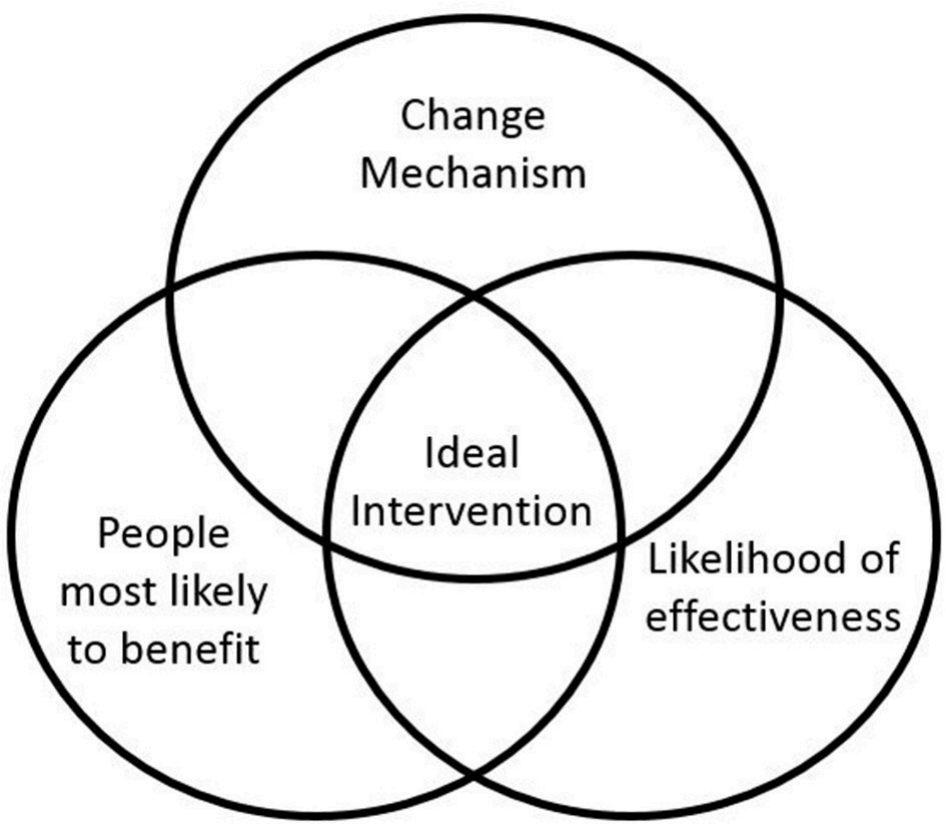
Graphic to aid discussion about the trial population.

Defining the eligibility criteria potential participants must meet to join the study is an important decision as it determines the group of people to whom trial results may be generalized. There was a structured discussion at the first investigator meeting about targeting the peer support intervention. The chief investigator leading the meeting used a graphic to launch the discussion in which the central zone refers to the ideal targeting of the intervention as an intersection of (1) people most likely to benefit (i.e., where need is highest), (2) existing evidence about what is most likely to work best, (3) what is known about how peer support works (the change mechanism). Figure 1 is a smarter version of the original graphic used in the meeting.

Concern was expressed by clinical academics around the table that if the trial showed a very weak or not statistically significant effect of peer support because we had not chosen the trial population carefully enough then we would do a disservice to peer support services (as these might be less likely to be commissioned going forward). Minutes from the meeting show that concern was raised that:

A negative result would have the potential to undermine peer support work as it is. (minutes)

Members of the team with lots of experience working on randomized controlled trials argued that a discrete rather than heterogeneous diagnostic population be chosen to ensure the methodological quality and subsequent impact of the trial on treatment guidelines:

The trial is not powered for subgroup analysis and if the result turns out to be negative then it will not be recognised in the international community nor adopted by guidelines … a specific population [should be] chosen, for example psychosis, for inclusion criteria. (minutes)

The two service user researchers on the team at that time and other members of the team involved in delivering peer support argued strongly against restricting inclusion by diagnosis on the grounds that peer support was not usually provided like this in practice and that the connections peers made were generally not on the basis of shared diagnosis. Minutes from the discussion record the following comment:

The essence of peer support must not be compromised and the peer support service developed and tested for the research must be one that is workable and justifiable to inpatient peers and peer workers in practice. Reasons for peer support being offered to one person over another have to be credible in the real world. Peer worker and peer support leader members of our team must be included in this design decision, it can't be made simply for research trial success purposes. We have not set out to design a diagnosis specific peer support service, we have set out to offer people support with discharge from inpatient to psychiatric care. Diagnoses can shift with each progressive encounter with secondary care and are culturally bound and are imposed and often not owned by the service users or peers themselves. We should be asking who the peer support would be most useful to. (minutes)

In later discussions about how a suitable population might be identified, clinical academics responding by suggesting an approach based on known predictors of our primary outcome (psychiatric readmission within 1 year of discharge):

…identifying people most likely to be readmitted, using numbers of readmissions as a predictor. (minutes)

It was agreed by the team that this approach would satisfy both the need for a discrete population that findings could be generalized to (ensuring methodological quality) while also retaining the integrity of peer support (by not defining peer relationships by diagnostic categories). A plan was put in place to approach trial sites for data about their inpatient populations so that a final decision could be made on eligibility criteria that would also enable us to feasibly deliver recruitment targets.

#### Reflections About the Trial Population

Three service user researchers who co-authored the paper reflected on their experience of applying the eligibility criteria in practice. It was noted that not using diagnosis as the main eligibility criteria supported researchers in working from a service user researcher perspective:

The non-diagnostic approach to recruitment fits well with the service user researcher approach to recruitment (e.g., being alongside someone as they considered whether to join the study, and communicating what peer support is). Overall, this has been a positive way of working - the reduced focus on diagnosis felt less discriminatory, medicalised, or pathologising. We appreciated the fact that it doesn't disclose to other service users on the ward the diagnosis of our participants or potential participants. (reflection)

The text above demonstrates a good amount of agreement between the perspectives of the service user researchers and the perspectives of others with lived experience who had been involved in the study at the time when this decision was made. In this example, although this was a decision that was taken before many of the service user researchers joined the project, the coproduced decision fitted well with the service user researchers' feelings about the “ethos” of the project.

### Trial Psychometric Measures and Outcomes

All trials define in advance a primary outcome used as the main measure to determine whether the intervention had an effect. The primary outcome is also used to calculate the necessary sample size for the trial. In addition, it is common to select a number of secondary outcomes, for which it is expected that the intervention will also have an effect. The primary outcome for this trial—psychiatric readmission within 12 months of discharge—had been suggested by the research funder, at review of the funding application, as an example a concrete indicator of patient benefit they would expect as the primary outcome of the trial. As such we note an absence of wider co-production across the team about the selection of the primary outcome.

The secondary outcomes were selected at two meetings as part of the development of the proposal. The meetings included academics and clinicians involved in evaluating peer support and conducting trials, as well as people with experience of developing, delivering and evaluating peer support services from a lived experience perspective. These discussions were informed by a peer support change model developed by the team based on previous qualitative research (Gillard et al., 2017), which had been coproduced by service user researchers, peer workers, clinicians and researchers. The change model suggested that peer support had an effect on hope, experience and anticipation of stigma, strength of therapeutic relationship and social connectivity.

There was no measure of clinical severity in the original proposal but a measure of clinical severity—the Brief Psychiatric Rating Scale (Overall and Gorham, 1962)—was added as a secondary outcome as a direct consequence of the decision not to define the population by diagnosis. This decision was taken by the chief investigator on the advice of members of the Trial Management Group with experience running randomized controlled trials in order to be able to describe, clinically, an otherwise diagnostically heterogeneous population. This was done largely so that the trial would meet peer review expectations for inclusion in high impact journals and systematic reviews, as well as to enable comparison with other peer support trials. The minutes of several meetings of the Lived Experience Advisory Panel document the reactions of members of the team to implementing this decision. There was an extended discussion about the use of the Brief Psychiatric Rating Scale at a Lived Experience Advisory Panel meeting held shortly after recruitment to the pilot trial began. It was noted that:

The service user researcher team have been arguing against doing it since this came to light as they started preparation for the data collection part of their role … the solution has been for service user researchers to decide how they will do this measure, do it openly, change some of the language of the tool and explain that they are rating it on the basis of conversation with the service users they are interviewing ‘peering through the psychiatric lens together'. (minutes)

And that:

The concerns service user researchers raised included using the wrong measure for peer support - clinical symptom change is not a particular aim of peer support; the potential for distress to service user researcher or participant due to similarity with previous experiences of psychiatric assessment, non-service user researcher/service user friendly language and administration, challenge to service user researcher role - service user researchers employed for lived experience perspective being asked and trained to take a psychiatric perspective. (minutes)

However it was also noted by the Lived Experience Advisory Panel that:

Co-production is maybe about being willing to keep going together and not “strutting out” when things don't fit with our ideas. (minutes)

Nonetheless, Lived Experience Advisory Panel meeting notes report the need for caution in interpreting the data produced by Brief Psychiatric Rating Scale:

We need to take care in our interpretation of the Brief Psychiatric Rating Scale data. Since if peer support goes well service users could be more open to sharing difficulties with the service user researcher at the second assessment; this could be (mis)interpreted as peer support making people worse. (minutes)

There was some discussion of the way in which delivery of the Brief Psychiatric Rating Scale had been adapted by the service user researchers:

The Brief Psychiatric Rating Scale would have damaged rapport if it had come anywhere but the end of the interview but service user researchers are happy with the way they have been able to make it more person friendly and be open about what it is. [Only] one participant so far shut down the interview at this point (i.e., when the Brief Psychiatric Rating Scale questions were asked). (minutes)

As a result of these discussions changes were made to the trial database to enable service user researchers to omit the Brief Psychiatric Rating Scale if it wasn't completed due to, for example, objections or distress from the participant.

#### Reflections About the Trial Psychometric Measures and Outcomes

Service user researchers who were co-authors of this paper reflected on the decision to include the Brief Psychiatric Rating Scale as an outcome measure. These reflections tell us about how co-production works in practice with respect to the consequences of the decision to include this very clinical measure. One described the initial plan of how to conduct this part of the interview as follows:

In practice, using the Brief Psychiatric Rating Scale means being transparent with service users; we are able to be honest about the very psychiatric approach of the Brief Psychiatric Rating Scale which does not necessarily fit with many aspects of the ethos of the project. (reflection)

This means taking a stance toward the measure which makes it clear that we wouldn't use this type of medical approach to understanding psychological distress as a first preference. Co-author service user researchers reflect that, despite approaching the issue as described above, it can still be difficult to conduct this assessment:

We often spend a lot of time building rapport with a participant, talking about lived experience, and working hard to create a safe, supportive environment, attempting to equalise inequalities in power. Using such a psychiatric measure abruptly changed the dynamics we had worked hard to create with the participant causing a sense of unease due to appearing contrary to the principles of survivor research, and can be uncomfortable to conduct from a service user perspective. This becomes even more difficult at follow up. At follow up, the Brief Psychiatric Rating Scale has to be scored in the middle of the interview to prevent scoring whilst un-blinded which means completing it in front of participants. (reflection)

Beyond the way that the rating scale affects the interviews with participants, there are additional issues with the use of the scale, as outlined in the following reflection:

The Brief Psychiatric Rating Scale is quite skewed toward psychotic type symptoms. Consequently, we have often felt the measure does not reflect the state of someone's emotional wellbeing accurately as, for example, there are only two or three places in which trauma or suicidality may affect the score. We have quite lengthy discussions with service users, sometimes spending 3 hours with each person, and it seems reductive to then use a number score on the Brief Psychiatric Rating Scale as this does not wholly capture the wealth of information communicated. (reflection)

The above statement communicates a frustration that can often be felt by researchers with a qualitative interest when they conduct semi-structured interviews which are used to generate numerical scores in quantitative research. The statement again reflects that this way of summarizing the trial population does not sit easily with many of the service user researchers, and would not be their first choice of method. The reflections continue:

Most of the service user researchers were not involved in the decision making process around using the Brief Psychiatric Rating Scale as we were not in post then, meaning there has been a lack of opportunity to coproduce in this area. This issue is aggravated as the research is a randomized controlled trial, for which the protocol needs to be fixed early on in the project. It is not possible to adapt many things as time goes on, unlike with our qualitative interview schedules which have been reworked and adapted after the pilot quite significantly, and have afforded plenty of opportunity to coproduce. (reflection)

This scenario, in which research decisions are coproduced, but many of the team of service user researchers did not have the opportunity to be involved in that co-production, seems likely to be commonly experienced in the context of randomized controlled trials. This is because these decisions are made well in advance of the start of data collection. The fact that the service user researcher jobs are advertised as working on a coproduced project can therefore raise unrealistic expectations of influencing key decisions. The chief investigator of the trial also reflected on the decision around including a clinical measure, illustrating how tension might remain long after a decision is taken:

I had misgivings about using Brief Psychiatric Rating Scale in the trial. All our other research has suggested that peer support works socially, it's about relationships and connections, rather than clinically. We didn't expect peer support to have an impact on clinical outcomes when we were developing the study, and that is also what the literature tells us. I knew that there would be a tension between the very clinical nature of the measure and the values underpinning peer support and service user research. However I could see the rationale for using a clinical measure to describe the trial population so that we would be able to say, if the peer support works, who it works for. We know that people are discharged from hospital when they are more or less well depending on very transient things like demand for beds and variations in how services are set up locally, so we did need something else here or it would be difficult for people to draw any conclusions about how relevant any findings from ENRICH would be in different parts of the UK or in different countries. (reflection)

In the following text, reflections about alternatives to the Brief Psychiatric Rating Scale, alongside alternative ways that this part of the project could have unfolded are explored:

Perhaps including a clinical measure was an executive decision I would have always felt I needed to make but there were consequences of not including the wider team, and especially service user researchers in that decision. First, we might have identified a different measure that could have been less challenging to use, which might have addressed many of the issues raised by the (service user researcher) team, issues that they had to work with on a daily basis. Second, had people felt involved in the decision, even if they had disagreed with it, then the subsequent discussion around how to implement Brief Psychiatric Rating Scale might have felt more collaborative and have been more productive of valuable learning about how best to measure things like ‘severity'. And finally, all our discussions were tinged by the decision having been already made, and then guillotined anyway as we needed to register the trial, which wasn't good for our sense of working co-productively as a team. (reflection)

The text above demonstrates the difficulty of coproducing whilst using a research method for which many things need to be specified in advance. The importance of planning plenty of time to coproduce at the stages of the project in which key decisions are made are highlighted, alongside the difficulty of trying to coproduce with people who were not on the project at a time when key decisions were made.

### Trial Statistical Analysis Plan

It is best practice in a trial to publish a statistical analysis plan demonstrating that analyses have been specified before outcomes data have been viewed (rather than analyses conducted to fit the data). The primary analysis was specified in the protocol, but additional analyses where the wider team might have meaningful input, included identification of groups for any subgroup analysis and specification of the Complier Average Causal Effect (CACE) analysis (see below).

Primary trial analyses examine “intention to treat”; i.e., all participants randomized to receive the intervention—whether they took up the offer or not—are compared with all participants randomized to care as usual (White et al., 2011). This is because people do not always take up treatment offers. We can additionally examine the effect of treatment receipt using a CACE analysis. A definition of what constitutes having received the intervention is needed; a minimum level of engagement with the intervention, below which participants can be assumed to have had no benefit. Subgroup analyses explore whether there was any difference in effect of the intervention for different groups of participants (e.g., men and women).

These analyses were initially discussed at meetings of the investigator team and the Trial Management Group where it was decided that the input of the Lived Experience Advisory Panel and service user researcher team was needed to inform these decisions. One of the trial statisticians held workshops with the Lived Experience Advisory Panel and service user researcher team; co-facilitated by the chief investigator. These meetings introduced and explained the statistical analysis plan as a whole, indicating where and why certain analyses had already been decided, and where there was scope for more co-production. Within the service user researcher team, two members had particularly valuable prior experience for this discussion, one from working in epidemiology and one from PhD research with a high degree of statistical content.

Discussions on the CACE analysis included “how many contacts” between participant and peer worker were enough to benefit from the intervention; the statistician and chief investigator suggested a single contact is potentially beneficial. However, notes from the service user researcher meeting indicate, based on their experiences of working on the trial, that:

First session with peer worker often focuses around logistical aspects of relationship and if this is the only session to occur it is unlikely that therapeutic benefit will have been delivered. (minutes)

Furthermore, service user researchers reminded the meeting of the importance of choice in developing peer support relationships, suggesting that:

A session in the community indicates that the participant has chosen to engage with the peer worker. Whilst in hospital participant may be visited by peer worker without them actively deciding to engage. (minutes)

From these discussions it was decided that the threshold for receipt of intervention for the CACE analysis is at least two contacts with peer worker, at least one of which should be in the community post-discharge. The discussions with both Lived Experience Advisory Panel and service user researcher team about potential subgroup analyses were wide-ranging. The statistician suggested to both meetings that we consider either a single subgroup analysis of a small number of study outcomes, or a small number of subgroup analyses of just the primary outcome. Both Lived Experience Advisory Panel and service user researchers felt that the latter option would be more useful as their experiences of involvement in and researching peer support suggested that there were a number of meaningful relationships between group identity and the way in which peer support might work.

The draft plan prepared by the statistician included broad diagnostic groups (psychotic disorders, personality disorders, and other non-psychotic disorders) as potential subgroups. The service user researchers had reservations about this due to the transitory nature of many participants' diagnoses and because they felt diagnoses did not always match the way participants described their own experiences:

Participants may have other diagnoses or may have a primary diagnosis that is not the cause of the current admission. We collect other data on medical history via self-report. This data may be challenging to use, it is collected as free text, includes diagnosis as described by participants that might not use standard terminology. (minutes)

The chief investigator argued in favor of keeping broad diagnostic category in the analysis because, irrespective of the validity of a diagnosis, people receive different treatment depending on their diagnosis which may interact with their experiences of peer support. Thus, diagnostic group was retained as a subgroup with the qualification that we would interpret any findings in relation to people's experiences of using different mental health services, rather than evidence that “peer support worked for some diagnoses, but not for others.”

Finally the Lived Experience Advisory Panel suggested that the mechanisms of peer support are in large part social—enabling people to connect to community—and so might work differentially in people who were already well connected socially compared to people who were isolated in the community, a suggestion which was well received and has been adopted to define suitable subgroups.

#### Reflections on Developing the Trial Statistical Analysis Plan

The statistician who led the workshops on the statistical analysis plan reflected on the involvement of service user researchers and the Lived Experience Advisory Panel in developing elements of the plan.

The input from service user researchers and the Lived Experience Advisory Panel was vital in deciding what assumptions were reasonable to make in carrying out this analysis. The decision to carry out an analysis using a binary cut off as to whether treatment was received did not address all the questions the service user researchers and Lived Experience Advisory Panel would have liked to ask. Other questions considered included what the effect would be of receiving only one session, or in response to different numbers of sessions. As a statistician I felt that I needed to guide the group away from more complex questions as I felt that limitations imposed by the study design and methods available would not allow us to get good answers to these questions. (reflection)

Service user researchers working on the project were positive and enthusiastic about their input into the statistical analysis plan and felt that co-production had worked really well in this area, as the following text demonstrates:

I felt that, from the start, the statisticians on the project were really interested in, and supportive of, incorporating ideas from members of the team with lived experience into the analysis plan. Co-production felt really well planned, smooth and organized. We had the opportunity to ask all the questions we'd like to about the plan, which were answered in full. Our suggestions were really well received and had a big impact on the plan. Co-production worked really well for all parties involved. (reflection)

In the above texts, the practice of combining statistical knowledge with clinical knowledge (from clinicians or those with lived experience), to inform the statistical methods used and the way those methods are applied is illustrated. Statisticians specializing in analysis of randomized controlled trials always work in interdisciplinary teams, drawing on the knowledge of others in the team to develop the statistical plan. Perhaps for this reason, developing a coproduced trial statistical analysis plan was one of the easier areas in which to coproduce the research.

## Discussion

This paper set out to explore the possibilities and challenges of coproducing randomized controlled trials, focussing on a randomized controlled trial of a peer support intervention in mental health which explicitly set out to coproduce knowledge and employed service user researchers. We illustrated this through examples of where co-production seemed to go well and where it was challenging, covering the three key areas of: identifying our trial population, creating a trial statistical analysis plan and selecting psychometric outcome measures. The decision around identifying our trial population involved a wide range of team members who also very explicitly identified the perspectives they were working from, as clinical academics, trialists, and service user researchers, and how that informed the views they brought to the discussion. The importance of not just including different perspectives, but of being explicit about those perspectives has been identified as key to the co-production of knowledge (Gillard et al., 2012a) and indicative of the social accountability of the knowledge production process (Nowotny et al., 2001). As such, we did not find that the knowledge of our research professionals was valued over the experiential knowledge of other team members (see also Scholz et al., 2017). The service user researchers' lived experience was valued as primary expertise on peer support and as such contributed in equal measure to our final decision about the trial population, alongside methodological insight. Service user researchers and team members involved in peer support took on a leadership role in advocating forcefully, at the outset of the project, that the essence of peer support not be undermined by the study design. Roper et al. (2018) indicate that having the means to take a leadership role in this way, especially for non-conventional research team members, characterizes non-tokenistic peer support. The initial research team meeting began with a discussion about research co-production, and team members were invited to describe in some length their role in the research and what they felt their priorities for the project were. Perhaps this approach, alongside the fact that nearly half the members of the research team were working from a lived experience perspective, enabled people to take on this leadership role. In this respect, at this stage of the project, co-production had been sufficiently resourced (Lambert and Carr, 2018).

As evidenced in the discussions around the statistical analysis plan and in the reflections of the statistician, we neither found that lived experience was devalued as a source of knowledge (Scholz et al., 2017), nor did we encounter reservations about the abilities of service user researchers to hold educated positions on the technical issues raised by the plan that have been cautioned against elsewhere as a barrier to co-production (Happell et al., 2015). Perhaps that was a reflection of the research literacy and expertise of our team. However, as identified by Roper et al. (2018), we did see open acknowledgment of power differentials that existed in the team in this phase of the study. The statistician was, in effect, the arbiter of what potential changes could be made to the plan, and made it very clear at the outset of both meetings where aspects of the analysis had already been determined—and why, methodologically, that needed to be the case—and where there remained meaningful opportunities for the analysis process to be shaped. This approach was appreciated in feedback from both the Lived Experience Advisory Panel and members of the service user researcher team.

We note how the co-production of decisions around trial population and statistical analysis plan had been made possible by the retention of a certain amount of flexibility in the research process. As regards selecting psychometric outcome measures, this flexibility was absent from the decision about including the Brief Psychiatric Rating Scale—it was presented as a fait accompli—and so limited opportunity for co-production. It is clear from the discussions referred to above that the team worked hard to address the challenges raised by the inclusion of the Brief Psychiatric Rating Scale but these efforts were imposed on the team *post-hoc*, rather than engaged in from the outset as a collaborative endeavor. In these circumstances co-production could be described as tokenistic, limited by the power imbalance in the team that flowed from that executive decision (Rose et al., 2010), especially for those service user researchers who came later to the team. Indeed wider members of the team had not been privy at all to that particular decision (Roper et al., 2018) and it is possible that the undermining of trust in the research-participant relationship that is referred to in the service user researcher reflections mirrors a damage to trust in the team at this point.

## Implications

On balance we reflect that it is possible to incorporate a co-production approach to research—as defined in Box 1 above—into a randomized controlled trial, especially with respect to the role of service user researchers in the research team. However, we also note that there are multiple challenges that need to be addressed to optimize co-production across all aspects of the project. Clarity around which aspects of decisions can be coproduced is essential, as is clear communication of the knock-on implications of any decision for the rest of the project. Our findings suggested that, in a randomized controlled trial, the methodology demands that co-production is front-loaded wherever possible as it could be challenging for service user researcher members of the team to implement some research decisions into practice where they had not been involved in early decision-making. This means most of the time for co-production must be scheduled toward the start of the project. However, we also found that co-production of the trial analysis strategy worked well within circumscribed and well-communicated limits.

## Recommendations

Scheduling time to co-produce decisions and recognize that much of this needs to be done at the planning (grant application) stages and early in the trial is recommended. It is likely that researchers will find that co-production occurs more or less completely in different areas of the project. We recommend reflecting on and documenting this to build the literature of the barriers and enablers of co-production in randomized controlled trials so that all interested parties can develop their skills and plan to coproduce research. Co-production of the analysis strategy, with clear explanations of the implications of the questions and the scope for co-production is recommended and can be very successful.

The expectations for influencing the methodology of the research in co-production can be high for all parties. Many contrasting views can be presented and not all these views can always be incorporated into the research. Team members can be very committed to the positions they bring to the research and expectations are not always met. We recommend regular reflection on the impact of co-production to support emotional well-being, morale and team cohesion.

Power differentials are always present in teams but creating an environment in which they can be honestly acknowledged and challenged when discussing co-production enables those in less powerful positions to have an impact on high value decision-making. Co-production adds time to a research project, which must be costed appropriately. Co-production in some aspects of a large research project, despite best intentions, may become tokenistic if those conditions are not met. Co-producing can be considered to be an additional variable when assessing quality of research (Sweeney et al., 2019).

## Strengths and Limitations

The study is limited by the constraints imposed by our approach to capturing and analyzing data about the research process. We rely on minutes from team meetings and the written first person reflections of team members as our case study data. Not all co-production takes place in team meetings and neither is co-production confined to decision making, while minutes and reflections do not necessarily include the views and experiences of all team members (arguably our data is somewhat selective). An ethnographic approach to exploring co-production and the randomized controlled trial, comprising observations and interviews conducted by a researcher who was not a team member might have offered a more comprehensive data set and a more systematic approach to analysis. However, as an initial exploration our approach offered feasibility. A focus on decision making, critical reflection on the inclusion of the diversity of voices in the team in those decisions, and consideration of the implementation of those decisions into practice does offer meaningful insight into key aspects of co-production (Gillard et al., 2012a). We were careful to select a range of positive and challenging experiences of research decision making and our findings were given external validity by our reading of the co-production literature.

## Conclusions

Co-production challenges and potentially changes aspects of randomized controlled trial methodology through the inclusion of a wider range of voices in the research-decision making process, including non-traditional expertise such as the lived experience of people who have used mental health services. Through balancing all the factors relevant for a decision, contributed by all the experts (methodological, clinical, and experts by experience), randomized controlled trials can be conducted in a way which incorporates and values service user perspectives, delivering research with greater social accountability which is also hopefully of higher quality and more relevant to service users and their mental health journeys.

## Data Availability

The datasets generated for this study are available on request to the corresponding author.

## Ethics Statement

This study was carried out in accordance with guidance including but not limited to, the Human Rights Act 1998, the Data Protection Act 1998, the Human Medicines Regulations 2012, ICH GCP, the World Medical Association Declaration of Helsinki, the NHS Research Governance Framework for Health and Social Care. The protocol was approved by the NHS Health Research Authority (HRA) on 19/05/2016 and the HRA London Bridge Research Ethics Committee on the 10/05/2016. All subjects gave written informed consent in accordance with the Declaration of Helsinki.

## Author Contributions

This is a coproduced paper. LG, SG, RM, and CM each produced first drafts of independent sections of this paper and reworked coproduced drafts of the paper. LG also co-ordinated wider coproduction for the paper. RM, GF, SG and LG wrote reflections. GF, MU, ML, and AS contributed feedback to drafts of the paper. All authors read and approved the final draft.

### Conflict of Interest Statement

The authors declare that the research was conducted in the absence of any commercial or financial relationships that could be construed as a potential conflict of interest.
